# Nirsevimab Uptake in a Pediatric Primary Care Network During the 2023-2024 RSV Season

**DOI:** 10.1001/jamanetworkopen.2025.20440

**Published:** 2025-07-14

**Authors:** Sarah Schaffer DeRoo, Tamima Hossain, Thevaa Chandereng, Jessica Lazerov

**Affiliations:** 1 Division of General and Community Pediatrics, Children’s National Hospital, Washington, DC; 2 Center for Translational Research, Children's National Hospital, Washington, DC.

## Abstract

This cohort study characterizes nirsevimab uptake among infants who did and did not receive the respiratory syncytial virus (RSV) vaccine during the 2023 to 2024 RSV season.

## Introduction

Nirsevimab, a monoclonal antibody, is highly effective in preventing respiratory syncytial virus (RSV)–related hospitalizations, giving it the potential to transform the landscape of RSV infections and their downstream impact on health care utilization.^1,2^ Despite its promise, nirsevimab uptake was variable in the US during its inaugural 2023 to 2024 RSV season, influenced in part by product shortages.^3,4,5,6^ Early evidence suggests that nirsevimab uptake was also associated with contextual and sociodemographic factors, including location and timing of nirsevimab administration, income, insurance type, parent employment status, and race.^3,4,5,6^ This aim of this study was to characterize nirsevimab uptake among eligible infants during the 2023 to 2024 RSV season.

## Methods

This retrospective cohort study was determined exempt by The Children’s National Hospital institutional review board, which waived the requirement for informed consent. We adhered to the STROBE reporting guideline. We characterized nirsevimab uptake among infants in a network of 5 outpatient, pediatric primary care clinics located in Washington, DC, during the 2023 to 2024 RSV season. Eligibility criteria included date of birth between February 19, 2023, and April 16, 2024, and 1 or more clinic visits between these dates. These dates captured all infants who were younger than 8 months when nirsevimab was available for administration. Data were extracted from the electronic medical record (EMR), including *Current Procedural Terminology* codes indicating nirsevimab administration (90380 and 90381), date of nirsevimab administration, *International Statistical Classification of Diseases and Related Health Problems, Tenth Revision (ICD-10)* codes indicating RSV infection (B97.4, J12.1, and J21.0), and date of RSV diagnosis. Demographic data were extracted from the EMR to identify potential differences in nirsevimab uptake that could be addressed using tailored strategies.

We calculated the rate of nirsevimab uptake and summarized sociodemographic characteristics using descriptive statistics. We employed χ^2^, Fisher exact, and *t* tests to demonstrate associations, comparing infants who did and did not receive nirsevimab. We employed univariable and multivariable logistic regression models to calculate odds ratios (ORs) to demonstrate associations with sociodemographic characteristics and identify interaction effects. The statistical significance threshold was a 2-sided *P* <.05. Analysis was conducted using R software version 4.4.3 (R Project for Statistical Computing). Refer to the eMethods in Supplement 1 for additional details.

## Results

Of 2162 eligible infants (1085 male [50.2%]), 831 (38.4%) received nirsevimab while 1331 (61.6%) did not. The mean (SD) age at nirsevimab administration was 1.8 (2.1) months. Sociodemographic variables are summarized in Table 1. Six infants (0.7%) who received nirsevimab and 25 infants (1.9%) who did not receive nirsevimab had a diagnosis of RSV during the study period.

**Table 1.** Characteristics of Infants Younger Than 8 Months Eligible for Nirsevimab During the 2023 to 2024 RSV Season

Characteristic	Participants, No./total No. (%)			*P* value^a^
	Overall (N = 2162)	Did not receive nirsevimab (n = 1331)	Received nirsevimab (n = 831)	
Sex				
Female	1077/2162 (49.8)	666/1331 (50.0)	411/831 (49.5)	.79
Male	1085/2162 (50.2)	665/1331 (50.0)	420/831 (50.5)	
Race				
Asian and Native Hawaiian or Pacific Islander^b^	44/2125 (2.1)	27/1306 (2.1)	17/819 (2.1)	.003
Black or African American	1565/2125 (73.7)	992/1306 (76.0)	573/819 (70.0)	
White	262/2125 (12.3)	135/1306 (10.3)	127/819 (15.5)	
Other^c^	254/2125 (12.0)	152/1306 (11.6)	102/819 (12.5)	
Unknown, No.^d^	37	25	12	
Ethnicity				
Hispanic or Latino	281/2118 (13.3)	164/1300 (12.6)	117/818 (14.3)	.26
Not Hispanic or Latino	1837/2118 (86.7)	1136/1300 (87.4)	701/818 (85.7)	
Unknown, No.^d^	44	31	13	
Insurance				
Commercial	432/2162 (20.0)	236/1331 (17.7)	196/831 (23.6)	<.001
Public	1444/2162 (66.8)	935/1331 (70.3)	509/831 (61.3)	
Self-pay	286/2162 (13.2)	160/1331 (12.0)	126/831 (15.2)	
Age at vaccine administration, mean (SD), mo	NA	NA	1.82 (2.10)	
RSV diagnosis	31/2162 (1.4)	25/1331 (1.9)	6/831 (0.7)	.03

Abbreviations: NA, not applicable; RSV, respiratory syncytial virus.

^a^ Pearson χ^2^ test.

^b^ Asian and Native Hawaiian or Pacific Islander were combined due to small sample size.

^c^ Reflects race listed as other or multiple in the electronic medical record.

^d^ Unknown excluded from Pearson χ^2^ test.

Race, insurance type, and RSV diagnosis were associated with nirsevimab uptake (Table 1). In the univariable logistic regression model, White race was positively associated with nirsevimab uptake compared with Black or African American race (OR, 1.63; 95% CI, 1.25-2.12; *P* < .001), while public insurance was negatively associated with nirsevimab uptake compared with commercial insurance (OR, 0.66; 95% CI, 0.53-0.82; *P* < .001) (Table 2). However, no sociodemographic factors were significantly associated with nirsevimab uptake in the multivariable logistic regression model, suggesting potential interaction effects. Only nonreceipt of nirsevimab was significantly associated with an RSV diagnosis in both univariable and multivariable regression models (OR, 0.38; 95% CI, 0.14-0.87; *P* = .03).

**Table 2.** Characteristics Associated With Nirsevimab Receipt During the 2023 to 2024 RSV Season

Characteristic	Univariable OR (95% CI)	*P* value	Multivariable OR (95% CI)	*P* value
Sex (n = 2162)				
Female	1 [Reference]	NA	1 [Reference]	NA
Male	1.02 (0.86-1.22)	.79	1.06 (0.89-1.26)	.54
Race (n = 2125)				
Black or African American	1 [Reference]	NA	1 [Reference]	NA
Asian and Native Hawaiian or Pacific Islander^a^	1.09 (0.58-2.00)	.78	0.91 (0.46-1.76)	.79
White	1.63 (1.25-2.12)	<.001	1.37 (0.95-1.98)	.09
Other^b^	1.16 (0.88-1.52)	.28	1.04 (0.73-1.49)	.82
Ethnicity (n = 2118)				
Hispanic or Latino	1 [Reference]	NA	1 [Reference]	NA
Not Hispanic or Latino	0.86 (0.67-1.12)	.27	0.89 (0.63-1.24)	.49
Insurance (n = 2162)				
Commercial	1 [Reference]	NA	1 [Reference]	NA
Public	0.66 (0.53-0.82)	<.001	0.77 (0.57-1.04)	.09
Self-pay	0.95 (0.70-1.28)	.73	1.12 (0.78-1.63)	.55

Abbreviations: NA, not applicable; OR, odds ratio; RSV, respiratory syncytial virus.

^a^ Asian and Native Hawaiian or Pacific Islander were combined due to small sample size.

^b^ Reflects race listed as other or multiple in the electronic medical record.

## Discussion

In this cohort study, nirsevimab uptake among eligible infants was 38.4%, which is low when compared with similar studies of commercially insured patients and where nirsevimab was administered in the inpatient setting.^3,4,5,6^ We identified important potential disparities in uptake that require further study. Infants who received nirsevimab were less likely to have an RSV diagnosis, suggesting that nirsevimab receipt decreased RSV-associated health care utilization. However, because this population is from a single geographic region, generalizability may be limited. Another limitation related to maternal RSV vaccination is that it is unknown how many infants in our cohort had RSV protection through maternal vaccination, which could impact our definition of eligible infants. Future efforts should attempt to identify barriers and facilitators of nirsevimab uptake to improve messaging and delivery during future seasons.

## Notes

Supplement 1.
eMethods.

Supplement 2.
Data Sharing Statement
