# Supplementary material for: Nirsevimab Uptake in a Pediatric Primary Care Network During the 2023-2024 RSV Season
Source: JAMA Netw Open. 2025 Jul 14;8(7):e2520440. doi: 10.1001/jamanetworkopen.2025.20440 (PMC12260984; doi:10.1001/jamanetworkopen.2025.20440)
Supplement: Supplement 1. — eMethods. [file jamanetwopen-e2520440-s001.pdf]

Supplemental Online Content

Schaffer DeRoo S, Hossain T, Chandereng T, Lazerov J. Nirsevimab uptake in a pediatric primary care network during the 2023-2024 RSV season. *JAMA Netw. Open*. 2025;8(7):e2520440. doi:10.1001/jamanetworkopen.2025.20440

eMethods.

This supplemental material has been provided by the authors to give readers additional information about their work.

© 2025 Schaffer DeRoo S et al. *JAMA Network Open*.

eMETHODS

We aimed to characterize nirsevimab uptake among eligible infants in a network of five pediatric primary care clinics during the 2023-2024 respiratory syncytial virus (RSV) season to inform messaging and delivery for future seasons. The clinic network began to administer nirsevimab to patients when the product became available on October 18, 2023, and ended administration on April 16, 2024 with the end of RSV season. Nationwide product shortages led the clinic network to develop a shared policy that prioritized infants at the highest risk for complications related to RSV infections, which we describe below. As such, we included in our analysis only infants who were under 8 months of age at any time during the 2023-2024 RSV season.

Pediatricians and nurse practitioners providing direct patient care recommended nirsevimab for all eligible infants during well and sick visits. The Centers for Disease Control and Prevention (CDC) Advisory Committee on Immunization Practices (ACIP) recommended that all infants <8 months old and <5 kg receive a single 50 mg dose of nirsevimab, and infants <8 months old and ≥5 kg receive a single 100 mg dose of nirsevimab. The ACIP recommended that certain high-risk infants and toddlers 8 through 19 months old receive a single 200 mg dose, administered as two 100 mg doses.

Due to product shortages early in the 2023-2024 RSV season, the clinic network developed a protocol to ensure equitable distribution of nirsevimab to infants and toddlers at the highest risk for RSV-related complications. The clinic network had sufficient supply of the 50 mg doses, which were administered to all eligible infants <8 months old and <5 kg according to the ACIP recommendations. The 100 mg nirsevimab dose was reserved for

© 2025 Schaffer DeRoo S et al. *JAMA Network Open*.

infants who were ≥5 kg and <12 weeks old and for certain infants ≥12 weeks but <8 months who were considered high risk. Approximately 20 patients aged 8 through 19 months qualified for the 200 mg dose and were prioritized to receive nirsevimab. Outreach encouraging families to schedule appointments solely for nirsevimab administration was limited due to concerns that the appropriate product might not be available at the appointment time. As supply improved, the clinic network changed its policy to include all eligible infants <8 months and children 8 through 19 months old deemed high risk per the CDC guidelines on February 20, 2024.

For the purposes of this study, infants were considered eligible for nirsevimab if they were <8 months old at any time during the 2023-2024 RSV season and had at least one lifetime clinic visit to establish care within the clinic network. Although a maternal RSV vaccine was newly available for pregnant persons, estimated national uptake rates were lackluster (17.8%, CDC data available from: https://www.cdc.gov/rsvvaxview/dashboard/2023-24-pregnant-persons-coverage.html), and maternal vaccination status was not available in our dataset. As a result, we were unable to account for maternal RSV vaccination status in this study.

Data were extracted from the electronic medical record (EMR) shared by the clinic network. Extracted data included demographic characteristics, including gender, race, ethnicity, and insurance type. Race categories extracted from the EMR included: American Indian or Alaska Native; Asian; Black or African American; Caucasian; Multiple; Native Hawaiian or Pacific Islander; Other Race; Patient Declined; and Unknown. Other race included those listed as other or multiple in the electronic medical record. Ethnicity

© 2025 Schaffer DeRoo S et al. *JAMA Network Open*.

categories extracted from the EMR included: Hispanic, Latino, or Spanish; Hispanic or Latino; Not Hispanic, Latino, or Spanish; Not Hispanic or Latino; Patient Declined; and Unavailable. Race and ethnicity were included to identify potential differences in nirsevimab uptake that could be addressed using tailored strategies.

The rate of nirsevimab uptake was determined by dividing the number of infants who received nirsevimab by the total number of eligible infants. Sociodemographic characteristics were summarized using descriptive statistics, including means and standard deviations for continuous variables and percentages for categorical variables. We employed chi-square tests and Fisher's exact tests if the expected number of events in the cells were less than 5 to demonstrate associations for categorical variables, and t-tests for continuous variables, comparing infants who did and did not receive nirsevimab. We used univariable and multivariable logistic regression models to model the rate of nirsevimab uptake and calculate odds ratios (OR) that are associated with sociodemographic characteristics. We employed additional univariable and multivariable logistic regression models to model RSV diagnoses and calculate odds ratios that are associated with sociodemographic characteristics, including nirsevimab receipt. Finally, we used multivariable logistic regression to look for interaction effects between sociodemographic characteristics.

© 2025 Schaffer DeRoo S et al. *JAMA Network Open*.
