# Supplementary material for: Nirsevimab Uptake in a Pediatric Primary Care Network During the 2023-2024 RSV Season
Source: JAMA Netw Open. 2025 Jul 14;8(7):e2520440. doi: 10.1001/jamanetworkopen.2025.20440 (PMC12260984; doi:10.1001/jamanetworkopen.2025.20440)
Supplement: Supplement 2. — Data Sharing Statement [file jamanetwopen-e2520440-s002.pdf]

Data Sharing Statement

Schaffer DeRoo. Nirsevimab Uptake in a Pediatric Primary Care Network During the 2023-2024 RSV Season. *JAMA Netw Open*. Published July 14, 2025.
doi:10.1001/jamanetworkopen.2025.20440

Data
Data available: No
